# SERS Detection of Dopamine Using Label-Free Acridine Red as Molecular Probe in Reduced Graphene Oxide/Silver Nanotriangle Sol Substrate

**DOI:** 10.1186/s11671-015-0937-9

**Published:** 2015-05-27

**Authors:** Yanghe Luo, Lu Ma, Xinghui Zhang, Aihui Liang, Zhiliang Jiang

**Affiliations:** Key Laboratory of Ecology of Rare and Endangered Species and Environmental Protection of Ministry Education, Guangxi Key Laboratory of Environmental Pollution Control Theory and Technology, Guangxi Normal University, Guilin, 541004 China; Hezhou University, Hezhou, 542899 China

**Keywords:** rGO/AgNT, Dopamine, Acridine red, Label-free, SERS

## Abstract

The reduced graphene oxide/silver nanotriangle (rGO/AgNT) composite sol was prepared by the reduction of silver ions with sodium borohydride in the presence of H_2_O_2_ and sodium citrate. In the nanosol substrate, the molecular probe of acridine red (AR) exhibited a weak surface-enhanced Raman scattering (SERS) peak at 1506 cm^−1^ due to its interaction with the rGO of rGO/AgNT. Upon addition of dopamine (DA), the competitive adsorption between DA and AR with the rGO took place, and the AR molecules were adsorbed on the AgNT aggregates with a strong SERS peak at 1506 cm^−1^ that caused the SERS peak increase. The increased SERS intensity is linear to the DA concentration in the range of 2.5–500 μmol/L. This new analytical system was investigated by SERS, fluorescence, absorption, transmission electron microscope (TEM), and scanning electron microscope (SEM) techniques, and a SERS quantitative analysis method for DA was established, using AR as a label-free molecular probe.

## Background

Noble metal nanoparticles have been widely employed in various fields such as catalysis, surface-enhanced Raman scattering, biomarkers, and thermal therapy due to their unique physical and chemical properties [1–4]. Especially nanogold and nanosilver have been of particular interest in spectroscopic analyses because of their intense visible-region absorption, which is largely attributed to the surface plasmon resonance. Compared to nanogold, nanosilver has the advantages of a low-cost, higher molar extinction coefficient [5] and stronger surface-enhanced Raman scattering (SERS) effects [6]. Moreover, nanosilver has a very broad range of antimicrobial activity [7] and represents a new generation of antimicrobials. The properties of metal nanoparticles depend strongly on the nanoparticles’ size and shape [8, 9]. Several methods have been developed for synthesizing nanosilver in a variety of shapes, including triangle, cube, rod, and wire [10–14]. But, most of the methods need high-concentration surfactants as stabilizer that restrains SERS effect and pollutes the environment. Thus, it is significant to explore a simple, stable, environmentally friendly, and highly SERS-active AgNT sol preparation, combined with new nanomaterials.

Graphene, with an atomically thin structure that consists of sp^2^-hybridized carbon atoms, has become a research hot spot in the field of physics and materials since it exhibits remarkable electronic, mechanical, and thermal properties [15]. However, the high specific surface area of graphene sheets makes them tend to form irreversible aggregation by van der Waals interaction, which restricts the large-scale application of graphene sheets [16]. Graphene oxide (GO) is a chemically treated graphene and has most recently emerged as a potential alternative to graphene. It is generally suggested that GO possesses various oxygenated functionalities such as hydroxyl and epoxy on the basal plane and carboxyl group at the edges [17]. The unique properties of GO such as superior molecule adsorbability, water solubility, fluorescence quenching ability, and Raman-scattering features make it suitable for biological applications [18–20]. Recently, the graphene-enhanced Raman scattering was discovered by Zhang’s group, and graphene has been shown to be an effective Raman-enhancement substrate [21, 22]. By combining the metal nanoparticle with graphene or graphene derivatives, a graphene-based composite has been fabricated for SERS analysis and better Raman-enhanced signals of the adsorbate were obtained [23–25]. Yang et al. [26] had researched the fabrication of water-dispersible Ag-GO composites by using tryptophan as a reducing and stabilizing agent and indicated that silver nanoparticles with spherical size were well dispersed on the surface of graphene oxide, and crystal violet exhibited excellent SERS activity in the synthesized Ag-GO nanocomposite substrates. Murphy et al. [27] had designed a reduced graphene-oxide-Ag nanoparticle composite to boost SERS sensitivity of a porphyrin derivative of TMPyP. Huang et al. [28] had described the preparation of Au nanoparticle-graphene-oxide composite; the developed approach offers well-controlled size, size distribution, and morphology of the metal nanoparticles in the metal-GO nanohybrids. They also demonstrated that the Au-GO composites are superior SERS substrates to the gold nanoparticles and exhibit significantly higher catalytic activities than the corresponding gold nanoparticles. Nowadays, the four main labeled technologies including radioactive-, enzyme-, fluorescence-, and nanoparticle-labeled technologies were applied widely to biochemistry analysis because of the advantages of high sensitivity and selectivity. High sensitivity and selectivity immunolabeling SERS and aptamer-labeling SERS technologies had developed based on the labeled technology combining with the immune and aptamer reactions [29–31]. As far as we know, the fingerprint of Raman spectroscopy could provide abundant information on molecular structure. Label-free SERS technique utilizing adequately the molecular fingerprint specificity can detect samples simply, rapidly, and nondestructively. Meanwhile, Raman spectroscopy has strong anti-photobleaching and is without water interference, and by the enhancement effect of metal nanoparticle substrate, label-free SERS techniques show considerable potential application in a biomacromolecule [32–35].

Dopamine (DA) is a catecholamine neurotransmitter associated with proper functioning of the central nervous system and is also an important marker for clinical disease; dopamine levels are connected to various pathologic states such as Parkinson’s or Alzheimer’s disease [36–38]. Therefore, the detection of dopamine is significant for medical or physiological research. At present, several methods including chemiluminescence [39], fluorescence spectrometry [40], high-performance liquid chromatography [41], colorimetry [42], electrochemistry [43, 44], and SERS [45–47] have been proposed for dopamine. SERS has been applied in fields of science and technology, such as surface adsorption, biochemical sensors, and biomedical analysis [48–50]. Oh et al. used an optofluidic SERS for label-free detection of dopamine molecules [45]. Bu et al. reported that AuNPs were used as substrate; a concentration of 1×10^−7^ to 1×10^−5^ mol/L DA can be detected by SERS technique [46]. To date, there are no reports about the label-free acridine red (AR) SERS for detection of dopamine in a reduced graphene oxide/silver nanotriangle (rGO/AgNT) sol substrate. In this paper, a new, simple, and rapid SERS spectral method has been proposed for detection of trace dopamine.

## Methods

### Materials

A 1.0×10^−4^ mol/L acridine red solution, 1.0 mol/L NaCl, 0.01 mol/L AgNO_3_, 0.06 mol/L trisodium citrate, 30 % H_2_O_2_, 0.1 mol/L NaBH_4_, pH 6.0 Na_2_HPO_4_-NaH_2_PO_4_ buffer solution, 0.1 % GO, and 1.0×10^−2^ dopamine hydrochloride were prepared. A 43.1 μg/mL silver nanotriangle (AgNT) was prepared as follows [51, 52]: in a triangle flask containing about 40.0 mL water, 2 mL 0.01 mol/L AgNO_3_, 3 mL 0.06 mol/L sodium citrate, 600 μL 30 % H_2_O_2_, and 600 μL 0.1 mol/L NaBH_4_ were added in turn with constant stirring for 15 min and diluted to 50 mL to obtain the AgNT sol; this mixture was then heated at 40 °C for 30 min to get rid of the excess H_2_O_2_ and NaBH_4_. The preparation of 43.1 μg/mL rGO/AgNT was the same as that of AgNT, except 5 mL of 0.1 % GO was injected into solution after the addition of NaBH_4_. All reagents were of analytical grade, and the water was highly pure sub-boiling water.

### Apparatus and Measurements

A model of DXR smart Raman spectrometer (Thermo Fisher, Waltham, MA, USA) was used with a laser wavelength of 633 nm and a power of 2.5 mW. A model of the F-7000 fluorescence spectrophotometer (Hitachi Co., Chiyoda-ku, Japan), a model of the TU-1901 double beam UV–vis spectrophotometer (Beijing Purkinje General Instrument Co., Ltd., Beijing, China), a model of FEI Quanta 200 FEG scanning electron microscope (FEI Co., Ltd., Eindhoven, Holland), and a model of SK8200LH ultrasonic reactor (Shanghai Kudos Ultrasonic Instrument Co., Ltd., Shanghai, China) were used.

### Procedure for SERS Detection of DA

A 400 μL 100 μg/mL rGO/AgNT, 35 μL 1.0×10^−4^ mol/L acridine red, and 10 μL 0.05 mol/L pH 6.0 Na_2_HPO_4_-NaH_2_PO_4_ buffer solution and a certain amount of DA were added into a 5-mL calibrated tube, diluted to 1 mL and mixed well. Then, 80 μL 1.0 mol/L NaCl was added and diluted to 2 mL. The SERS intensity at 1506 cm^−1^ (*I*) was recorded by a Raman spectrometer. A blank reagent (*I*)_0_ without DA was recorded, and the value of Δ *I* = *I* − (*I*)_0_ was calculated.

## Results and Discussion

### Principle

**Fig. 1:**
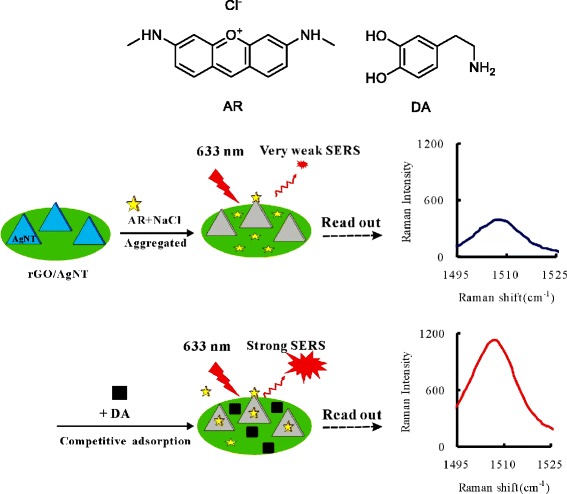
Principle of SERS detection of DA

Several groups have contributed to the knowledge that acridine orange (AO) can be bound on rGO to form an AO-rGO complex through electrostatic and π-π stacking interactions, resulting in the effective fluorescence quenching of AO [53, 54]. The structure of the AR molecule is shown in Fig. 1. AR is also a cationic dye with three aromatic rings and two amino groups in its chemical structure, and AO has a similar structure. The GO has some oxygen-containing functional groups, such as epoxy, hydroxyl, and carboxylic groups, which make GO in aqueous dispersion negatively charged, so cationic AR molecules can absorb on the GO surface through electrostatic interactions. Besides, the huge aromatic surface of GO can attract AR through π-π stacking. In the presence of a pH 6.0 PBS buffer solution containing 0.04 mol/L NaCl, when AR molecules were added into a rGO/AgNT solution, AR could absorb on the rGO surface by π-π stacking and electrostatic interactions, but absorption on the AgNT aggregate surface was so poor showing a weak SERS signal. As shown in Fig. 1, the DA molecule has an aromatic ring, two hydroxyl groups, and an amino group in its chemical structure. Upon addition of DA, it could also adsorb on the rGO sheets due to π-π stacking, and dopamine further interacts with the hydroxyl groups of rGO sheets due to multiple hydrogen bonding. Thus, DA molecules have a much stronger affinity to the rGO surface in comparison with AR molecules which was similar to the system of DA-Rh6G-GO [55]. When the concentration of rGO/AgNT held constant, the number of binding sites on the rGO surface was constant, AR and DA molecules competed for similar adsorption sites on the rGO surface. The adsorption of DA molecules can lead to desorption of AR molecules from the rGO surface; the desorbed AR molecules adsorbed on the AgNT aggregates and showed a strong SERS signal. When the concentration of DA increased, the desorbed AR molecules increased, and the AR molecules adsorbed on the AgNT aggregates surface increased gradually. The increased SERS intensity responds linearly with the concentration of DA. Accordingly to these, a SERS spectral method with label-free AR can be developed for the determination of trace DA in solution.

### SERS Spectra

**Fig. 2:**
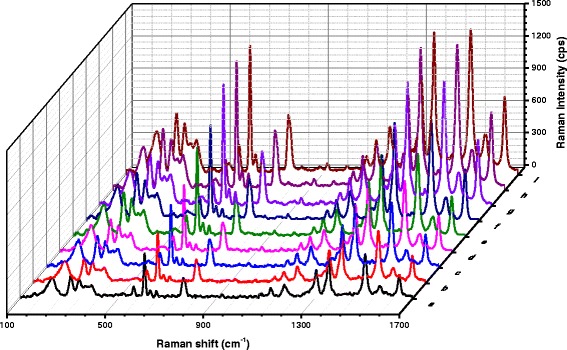
SERS spectra of the AR-rGO/AgNT-DA system. (*a*) 8.62 μg/mL rGO/AgNT-1.75×10^−6^ mol/L AR-pH 6.0 PBS-0.04 mol/L NaCl; (*b*) (a)-10 μmol/L DA; (*c*) (a)-75 μmol/L DA; (*d*) (a)-100 μmol/L DA; (*e*) (a)-175 μmol/L DA; (*f*) (a)-250 μmol/L DA; (*g*) (a)-375 μmol/L DA; (*h*) (a)-500 μmol/L DA; (*i*) 8.62 μg/mL AgNT-1.75×10^−6^ mol/L AR-pH 6.0 PBS-0.04mol/L NaCl

In the substrate of rGO/AgNT sol, AR exhibited SERS peaks at 608, 771, 1308, 1358, 1506, and 1646 cm^−1^, respectively. These peaks were ascribed to the outside surface deformation of CH, ring breathing, bending vibration of aromatic CH, stretching vibration aromatic CH, bending vibration of NH, and bending vibration of aromatic C=C, respectively. When AR was mixed with rGO/AgNT, AR adsorbed on the rGO surface due to electrostatic and π-π stacking interactions; only a small amount of AR molecules were absorbed on the AgNT aggregate surface, and the SERS signal was weak. Upon addition of DA, there were strong interactions between DA and rGO that resulted in the AR desorption from the rGO; the desorbed AR molecules adsorbed on the AgNT aggregates and showed strong SERS effect. The SERS intensity of AR-rGO/AgNT-DA system at 1506 cm^−1^ increased linearly as the concentration of DA increased (Fig. 2(a–h)). When 500 μmol/L DA was added, the SERS intensity of the system reached a maximum that was identical to the SERS intensity of AR on the AgNT aggregates without rGO (Fig. 2(i)). Based on the competitive adsorption between DA and AR, the original SERS intensity of AR was recovered, which corresponded with the principle.

### Fluorescence Spectra

**Fig. 3:**
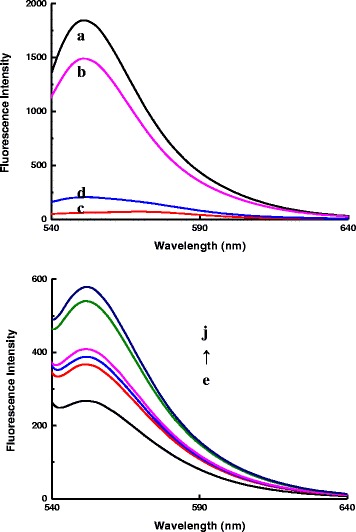
Fluorescence spectra of the AR-rGO/AgNT-DA system. (*a*) 1.75×10^−6^ mol/L AR-pH 6.0 PBS; (*b*) 1.75×10^−6^ mol/L AR-pH 6.0 PBS-8.62 μg/mL AgNT-0.04 mol/L NaCl; (*c*) 20 μg/mL GO -1.75×10^−6^ mol/L AR-pH 6.0 PBS; (*d*) 20 μg/mL GO -1.75×10^−6^ mol/L AR-pH 6.0 PBS -175 μmol/L DA; (*e*) 8.62 μg/mL rGO/AgNT-1.75×10^−6^ mol/L AR-pH 6.0 PBS-0.04 mol/L NaCl; (*f*) (a)-50 μmol/L DA; (*g*) (a)-100 μmol/L DA; (*h*) (a)-175 μmol/L DA; (*i*) (a)-250 μmol/L DA; (*j*) (a)-500 μmol/L DA

In the presence of the pH 6.0 PBS buffer solution, the characteristic emission peak of AR is located at 552 nm when excitation wavelength is 520 nm (Fig. 3(a)). When AR was mixed with AgNT aggregates, fluorescence intensity of AR remained strong (Fig. 3(b)). When AR was mixed with GO, AR was adsorbed on the GO surface by π-π stacking and hydrophobic interactions; fluorescence quenching of AR took place because of the photoinduced electron transfer or long-range resonance energy transfer between AR and GO; the strong fluorescence of AR was almost entirely quenched by the GO (Fig. 3(c)). After DA addition, the strong binding of DA with GO resulted in a release of AR from GO, so the fluorescence intensity was enhanced (Fig. 3(d)). Upon addition of AR to the rGO/AgNT solution, the fluorescence intensity was weak due to the adsorption of AR molecules on the rGO surface and the fluorescence quenching of AR. The added DA molecules led to the AR desorption from the rGO because the intermolecular interactions of DA and rGO were stronger than those in the case of AR and rGO; finally, the fluorescence intensity at 520 nm increased as the concentration of DA increased (Fig. 3(e–j)), which was in agreement with the principle.

### Absorption Spectra

**Fig. 4:**
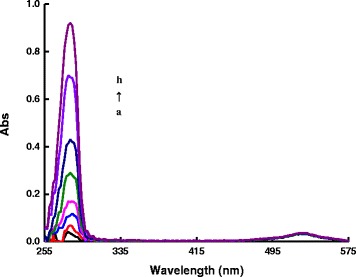
Absorption spectra of the AR-DA system. (*a*)1.75×10^−6^ mol/L AR-pH 6.0 PBS; (*b*) (a)-25 μmol/L DA; (*c*) (a)-50 μmol/L DA; (*d*) (a)-100 μmol/L DA; (*e*) (a)-175 μmol/L DA; (*f*) (a)-250 μmol/L DA; (*g*) (a)-375 μmol/L DA; (*h*) (a)-500 μmol/L DA

**Fig 5:**
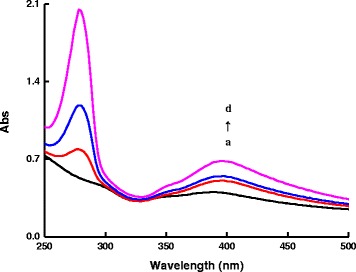
Absorption spectra of the rGO/AgNT-DA system. (*a*) 8.62 μg/mL rGO/AgNT - pH 6.0 PBS-0.04 mol/L NaCl; (*b*) (a)-100 μmol/L DA; (*c*) (a)-250 μmol/L DA; (*d*) (a)-500 μmol/L DA

**Fig. 6:**
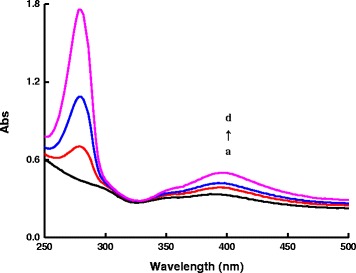
Absorption spectra of the AR-rGO/AgNT-DA system. (*a*) 8.62 μg/mL rGO/AgNT -1.75×10^−6^ mol/L AR- pH 6.0 PBS-0.04 mol/L NaCl; (*b*) (a)-100 μmol/L DA; (*c*) (a)-250 μmol/L DA; (*d*) (a)-500 μmol/L DA

There is an absorption peak at 528 nm for AR. The absorption peak at 280 nm was related to the benzene rings of DA, and the intensity was increased with increasing DA concentration (Fig. 4). In the rGO/AgNT-DA system, absorption peak of DA was still at 280 nm, there was a new absorption peak at 400 nm, and the intensity was increased with the increasing DA concentration that indicated the formation of a rGO/AgNT-DA conjugate (Fig. 5) and suggested that DA had stronger affinity to rGO. In the AR-rGO/AgNT-DA system, as the DA concentration increased, the rGO/AgNT-DA conjugates increased gradually, more and more AR molecules desorbed from rGO, the absorption peak at 280 nm was also increased, and absorption peak at 400 nm was increased with the increasing rGO/AgNT-DA conjugate (Fig. 6).

### Transmission Electron Microscope and Scanning Electron Microscope

**Fig. 7:**
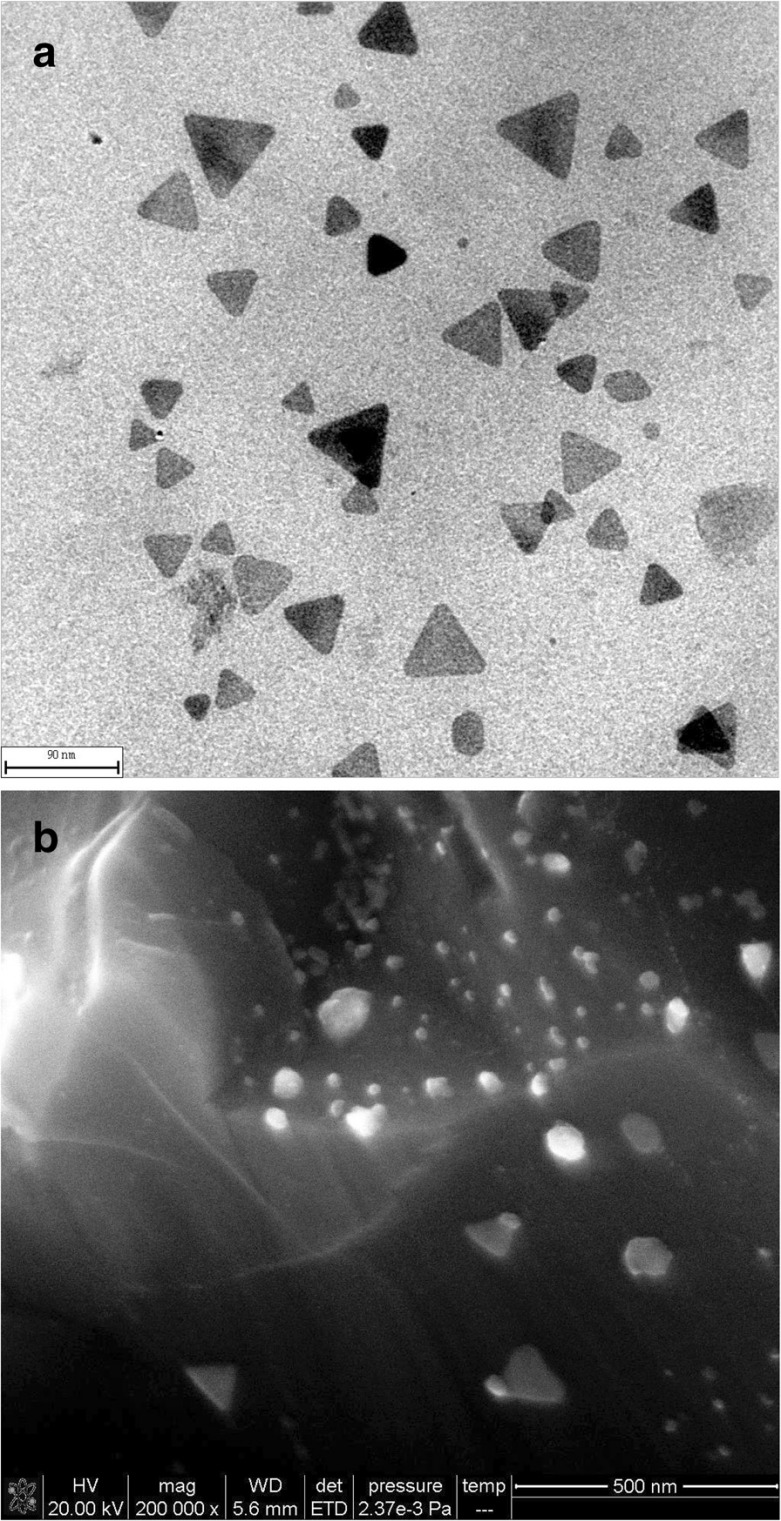
TEM of AgNT and SEM of rGO/AgNT. **a** TEM image of the 43.1 μg/mL AgNT; **b** SEM image of the 43.1 μg/mL rGO/AgNT

Silver nanotriangles were prepared by reducing AgNO_3_ with NaBH_4_ in the presence of H_2_O_2_ and sodium citrate. Figure 7a showed that most of the particles were silver nanotriangles with the side length between 10 and 70 nm. The rGO/AgNT composites were prepared by addition of GO; as showed in Fig. 7b, rGO with wrinkles were observed, and silver nanotriangles were adsorbed on the rGO surface.

### SERS Quantitative Analysis of DA

**Fig. 8:**
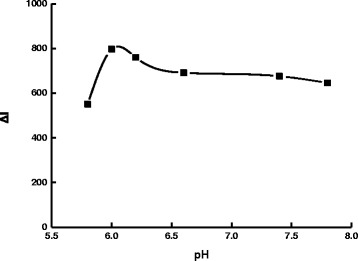
Effect of pH. 1.75×10^−6^ mol/L AR-PBS-2.5×10^−5^ mol/L DA-0.05 mol/L NaCl-5.39 μg/mL rGO/AgNT

**Fig. 9:**
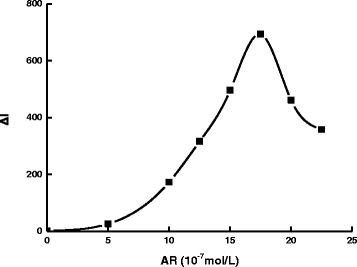
Effect of AR concentration. pH 6.0 PBS-2.5×10^−5^ mol/L DA-0.05 mol/L NaCl-5.39 μg/mL rGO/AgNT

**Fig. 10:**
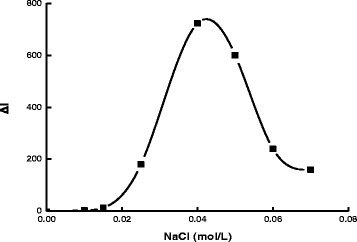
Effect of NaCl concentration. 1.75×10^−6^ mol/L AR-pH 6.0 PBS-2.5×10^−5^ mol/L DA -5.39 μg/mL rGO/AgNT

**Fig. 11:**
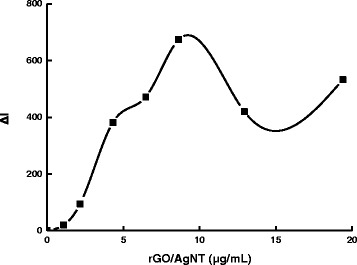
Effect of rGO/AgNT concentration. 1.75×10^−6^ mol/L AR-pH 6.0 PBS-2.5×10^−5^ mol/L DA-0.04 mol/L NaCl

The effect of pH for the PBS buffer solution was studied (Fig. 8). The Δ*I* value reached its maximum when the pH was 6.0. Thus, a pH 6.0 PBS buffer solution was selected for use. The effect of AR concentration on the determination was studied (Fig. 9). When the concentration of rGO held constant, as the concentration of AR increased, Δ*I* was increased as well. The concentration of AR was more than 1.75×10^−6^ mol/L; the SERS signal of the system without DA increased due to excess AR absorbed on the AgNT aggregates that resulted in the decrease of Δ*I*. Thus, a 1.75×10^−6^ mol/L AR was chosen for the assay. NaCl was a good aggregation reagent for AgNT. The effect of NaCl concentration on the determination was studied (Fig. 10). As the concentration of NaCl increased, Δ*I* was enhanced as well. When the concentration of NaCl was 0.04 mol/L, the Δ*I* value reached the maximum. When the concentration of NaCl increased further, the Δ*I* value decreased because the AgNT aggregated excessively and precipitated. Thus, a 0.04 mol/L NaCl was chosen for the assay. The influence of the rGO/AgNT concentration on the determination was studied (Fig. 11). When the concentration of rGO/AgNT was 8.62 μg/mL, the Δ*I* value was maximal. Thus, a 8.62 μg/mL rGO/AgNT was chosen for the assay.

**Fig. 12:**
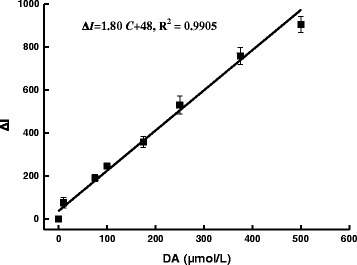
Working curve of the AR-rGO/AgNT-DA system. 8.62 μg/mL rGO/AgNT-1.75×10^−6^ mol/L AR-pH 6.0 PBS-0.04 mol/L NaCl-DA

For a quantitative method, a linear relationship exists in a fit range that is a linear range. For this SERS quantitative analysis method, the SERS intensity at 1506 cm^−1^ (*I*) increased with the DA increase. In the absence of DA, the blank value (*I*)_0_ is about 388. When DA concentrations are lower than 2.5 μmol/L, the *I* values are very close to the blank due to the unclear adsorption difference for the low-concentration DA. When the DA concentration is higher than 500 μmol/L, the *I* values hold constant due to the DA-saturated adsorption on the rGO/AgNT. When DA concentration (*C*) is in the range of 2.5–500 μmol/L, the increased SERS intensity Δ*I* at 1506 cm^−1^ increased linearly, with a regress equation of Δ*I* = 1.80*C* + 48, *R*^2^ of 0.9905, and a detection limit of 1.2 μmol/L (Fig. 12). Each measurement for data points was determined five times; for the relative standard deviations of determination, different DA concentrations were in the range of 2.1–5.1 %; this showed the method is accurate. The analytical system also can be determined by DA absorption and fluorescence spectral techniques (Table 1), and the SERS method is most sensitive with the biggest slope. Thus, the SERS method was chosen for detection of DA. The influence of coexistence substances on the determination was examined within an error of ±10 %. Results (Table 2) indicated that common substances do not interfere with the determination of 100 μmol/L DA, and this method has good selectivity. The DA in dopamine hydrochloride injection samples with a reference concentration of 5.29×10^−2^ mol/L dopamine hydrochloride was determined by this SERS method. A 5-μL dopamine hydrochloride injection sample that was diluted five times was used for the determination of DA content, with a relative standard deviation (RSD) of 3.9–5.3 %. A recovery of 98.3–103 % was obtained when a known DA was added to the samples (Table 3).

**Table 1:** Analytical features of the three spectral methods

Method	Regress equation	Linear range (μmol/L)	Coefficient	Detection limit (μmol/L)
SERS	Δ*I*=1.80*C* + 48	2.5–500	0.995	1.2
Fluo^a^(Fig. 3(e–j))	ΔF_552nm_ = 0.59*C* + 51	50–500	0.923	25
Abs (Fig. 5)	ΔA_280nm_ = 0.0018*C*−0.03	25–500	0.996	12

*Abs* absorption method

^a^Fluorescence method

**Table 2:** Effect of coexistence substances

Coexistent substance	Tolerance concentration	Relative error (%)	Coexistent substance	Tolerance concentration	Relative error (%)
Glycine	5000 μmol/L	−2.0	Zn^2+^	500 μmol/L	7.0
L-phenylalanine	2000 μmol/L	8.0	Mg^2+^	200 μmol/L	2.0
Ascorbic acid	2000 μmol/L	6.0	Cu^2+^	250 μmol/L	−0.9
L-aspartic acid	125 μmol/L	0.5	Ni^2+^	250 μmol/L	−10.0
HSA	0.025 μg/mL	4.0	BSA	4 μg/mL	−6.0

**Table 3:** Results for the detection of dopamine hydrochloride in samples

Added (μmol/L)	Single value (μmol/L)	Average (μmol/L)	Recovery (%)	RSD (%)
0	28.4, 27.9, 25.9, 26.3, 27.5	27.2	-	3.9
25	54.3, 48.6, 53.5, 50.2, 55.1	52.3	103	5.3
75	100.0, 95.2, 98.4, 104.9, 106.1	100.9	98.3	4.5
200	231.2, 206.9, 232.0, 228.4, 229.2	225.5	99.2	4.7

## Conclusions

The rGO/AgNT composite sol was prepared for SERS substrate. When AR and DA were added into the rGO/AgNT sol, competitive adsorption occurred between DA and AR around the rGO surface; DA has stronger affinity to rGO than that of AR that resulted in desorption of AR molecules from the rGO surface and adsorption on the AgNT aggregate surface that exhibited strong SERS effects. The intensity of SERS peak at 1506 cm^−1^ increased as the concentration of DA increased. A new SERS method was developed for the determination of DA over 2.5–500 μmol/L based on the label-free probe.
